# Responses of Soil Microbial Traits to Ground Cover in Citrus Orchards in Central China

**DOI:** 10.3390/microorganisms9122507

**Published:** 2021-12-03

**Authors:** Yupeng Wu, Xue Wang, Ronggui Hu, Jinsong Zhao, Yanbin Jiang

**Affiliations:** College of Resources and Environment, Huazhong Agricultural University, Wuhan 430070, China; wyp19851205@126.com (Y.W.); wangxue5113@163.com (X.W.); rghu@mail.hzau.edu.cn (R.H.); jszhao@mail.hzau.edu.cn (J.Z.)

**Keywords:** ground cover, legume, soil properties, microbial community, plant-microbe interactions

## Abstract

A clear understanding of which factors play an important role in the development of the soil microbial community in orchards will benefit our understanding of ground cover impacts on soil nutrient cycling. Thus, in the present study, grass properties, soil properties, and soil microbial community structure were determined in a citrus orchard after 5 years of management with different types of ground cover (NG: natural grass, LP: monoculture of legumes, and NL: mixed culture of natural grasses and legumes) to evaluate how ground cover biomass and nitrogen-fixing ability drive soil physicochemical and microbial traits. Plant biomass carbon (BC) and nitrogen (BN) were significantly higher in LP and NL than NG and showed a significant (*p* < 0.01) positive relationship with soil total carbon (TC), NO_3_^−^-N (NN), and dissolved organic carbon (DOC) content. In addition, the amount of biologically fixed nitrogen (FixN) showed a significant positive relationship with soil total nitrogen (TN) (*p* < 0.05) and NH_4_^+^-N (AN) content (*p* < 0.01). We also observed a difference in the soil microbial community structure between plots with and without legumes. The TC and BN were the most influential factors driving bacterial and fungal communities, respectively. Nevertheless, FixN explained less than 9% of the differences in soil bacterial and fungal communities. Our results suggest that grass biomass and FixN are the strong drivers of soil chemical properties, whereas ground cover and soil properties both contribute significantly to the soil microbial community structure.

## 1. Introduction

Citrus trees are the most commonly planted fruit trees worldwide. The red soil regions of middle and south China represent the largest citrus cultivation area, with a crop area covering approximately 2.48 × 10^6^ ha according to the 2020 government statistics (China National Bureau of Statistic, http://www.stats.gov.cn/, accessed on 1 September 2020). Under the requirement of chemical fertilizer reduction by the Ministry of Agriculture and Rural Affairs, ground cover has been widely used in citrus orchards in China.

Ground cover is considered one of the most important management practices in orchard systems, improving soil physical and chemical properties [1,2] and enhancing soil microbial activities [3,4,5]. However, various types of ground cover perform different functions on the soil microbial community. For example, in persimmon orchards under long-term mulching, *Phenylobacterium* was enriched in *Chamaecrista rotundifolia* mulching soil while *Rhodoplanes* was enriched in *Arachis pintoi* mulching soil [6]. Similar results have been reported for apple orchard soil, where the ground cover significantly altered the soil bacterial community structure and function, and different cover species had different impacts [7]. However, still some studies have found that soil properties rather than ground cover species were the important factors in determining soil microbial community structure [8,9]. As soil microbial communities mediate critical ecosystem carbon (C) and nutrient cycles [10], a clear understanding of which factors play an important role in the development of the soil microbial community in orchards will benefit our understanding of ground cover impacts on soil C and nutrient cycling.

In China, various ground cover species are used in orchards, including natural grasses, crops, legumes, crucifers, and so on [11,12]. Regarding their higher biomass and nitrogen fixation ability, legumes are frequently intercropped in orchards, solely or with natural grasses, and form different types of ground cover models [11,13]. Orchard soil with legumes usually shows a higher nutrient content due to the higher amount of nitrogen (N) from biomass residues [14]. In addition, the litter of legume species is rich in N and decomposes readily, with positive impacts on soil biota [14,15,16]. Thus, legumes have more significant effects than grasses (both C_3_ and C_4_ grasses) both on soil properties and soil microorganisms [17,18]. What is currently not clear, however, is which factor, improved soil fertility or ground cover species, drives more on determining soil microbial community structure. The aim of the present study is to evaluate how ground cover biomass and nitrogen-fixing ability drive different soil physicochemical and microbial traits. We hypothesized that, under different ground cover models, (1) ground cover biomass returning results in distinct soil physicochemical properties and (2) nitrogen-fixing via ground cover more significantly determines the soil microbial community than soil properties.

## 2. Materials and Methods

### 2.1. Field Experiments

The study was conducted in a 15-year-old citrus (*Citrus reticulata* “Seedless Ponkan”) orchard at Dangyang in Yichang City, Hubei Province, China (30°39′48.98″ N, 111°48′24.82″ E, 79 m asl.). The area features a subtropical monsoon climate with 1850 h of sunshine per year, an annual mean temperature of 16.4 °C, and an annual precipitation of 936–1048 mm. The soil was classified as loam clay according to the USDA soil texture classification system. The planting row space of the citrus trees was 3 m × 4 m.

Prior to the present study (from 2004 to 2013), traditional clean tillage had been applied in the orchard. Ground cover management was started in 2013 and aimed at three different types of ground cover: natural grass (NG), legume monoculture (LP), and mixed culture of natural grass and legumes (NL). The legume species *Vicia villosa* var. *glabrescens* was sown at a seeding rate of 75 kg ha^−1^ in LP and 25 kg ha^−1^ in NL in October, whereas other grasses were naturally growing. The grasses were mown in May and left on the soil surface. Inorganic fertilizers at rates of 410 kg N ha^−1^ yr^−1^, 240 kg P ha^−1^ yr^−1^, and 381 kg K ha^−1^ yr^−1^ were divided into basal dressing and topdressing, which were applied in all experimental orchards in December (after fruit harvesting) and the following June, respectively. The N, P, and K fertilizers were applied as urea, calcium superphosphate, and potassium sulfate, respectively.

### 2.2. Sampling and Analysis

Three randomly replicated experimental plots with an area of 120 m^2^ (10 citrus trees in each plot) in each treatment were chosen. In each plot, grass investigation was carried out through a five-point (five 1 m × 1 m quadrats) sampling method at the early flowering stage in late April 2019. Plant coverage was measured based on the five quadrats in each plot and was represented by the ratio of the shady area of grasses to the total area of a quadrat. Species richness (SR) of grass communities was investigated in each quadrat, and subsequently, grasses were clipped 2 cm above the ground to obtain grass shoot. After removing the aboveground grasses, five root samples in each quadrat were drilled using a 7.5-cm-diameter root core, sieved (0.85 mm), and washed with tap water. The harvested grass shoot and root materials were oven-dried and weighted for obtaining the shoot biomass (SB) and root biomass (RB). The total carbon and nitrogen contents in the grass shoots and roots were detected by a C/N elemental analyzer (Vario-Max CN, Elementar, Germany). Then, the amount of grass biomass accumulated C (BC) and N (BN) were calculated through multiplying the grass biomass by the carbon and nitrogen contents, respectively.

The amount of biologically fixed nitrogen (FixN) by legumes was determined by the ^15^N natural abundance technique according to Boddey et al. [19]. Briefly, N_2_-fixing legume species were collected together with other non-N_2_-fixing species as reference plants. The oven-dried legume and non-legume plants were ground separately, and then analyzed for their total N and ^15^N contents by using the C/N elemental analyzer and an N isotope analyzer (Finnigan, MAT 253), respectively. The results are expressed as “delta” notation:

δ = (R_sample_/R_standard_−1) × 1000;

where R_sample_ and Rs_tandard_ are the ^15^N: ^14^N ratios of the sample and the standard (air), respectively. When the δ value of a legume in a plot was significantly different from the average δ value of all reference plants in that plot, the proportion of fixed N in the plant was calculated using the following formula:

%Ndfa = (δ^15^N_reference_− δ^15^N_legume_/(δ^15^N_reference_−B) × 100;

where δ^15^N_reference_ is the mean value of the δ^15^N of the reference species at each plot, δ^15^N_legume_ is the mean δ^15^N value for N_2_-fixing legumes, and B is the δ^15^N value for the fixing plants cultivated in the absence of a mineral N supply, which was assumed to be −1.24%. Subsequently, the amounts of biologically fixed N were estimated by multiplying the biomass of each plant in one plot by the average of the proportion of fixation of the species in the plot.

During the grass investigation, soil bulk density (BD) was determined by the cylinder ring method in each plot, respectively. In each plot, five soil subsamples (0–20 cm) were collected by circular soil collector from each quadrat, respectively, and then composited into one to represent the soil sample from this plot. These soil samples were subsequently divided into two subsamples: one was stored at 4 °C for the measurement of soil physicochemical properties, and the other was stored at −80 °C for soil microbial community assessment. Soil pH was estimated in a 1:2.5 soil-water mixture using a pH meter (PHS-3C, INESA, Shanghai, China). The parameters AN, NN, and dissolved organic carbon (DOC) were extracted with K_2_SO_4_ solution and detected using a flow-injection auto-analyzer (AA3, Seal, Norderstedt, Germany) and a TOC analyzer (TOC-VWP, Shimadzu Corporation, Kyoto, Japan), respectively. Soil dissolved organic nitrogen (DON) equals the total dissolved N (TDN) minus the inorganic N (the sum of AN and NN), where TDN was determined using alkaline persulfate digestion. Soil TC and TN contents were also determined by using the elemental analyzer. Soil microbial biomass C (MBC) and N (MBN) were analyzed using the chloroform fumigation extraction method [20].

To characterize the soil microbial communities, we extracted and sequenced the total genomic DNA of soil samples. The DNA was extracted from 0.5 g fresh soil using the PowerSoil DNA isolation kit (MoBio Laboratories Inc. San Diego, CA, USA). The quantity and quality of extracted DNA were checked by a NanoDrop2000 UV-VIS spectrophotometer (NanoDrop Technologies, Wilmington, USA). Subsequently, MiSeq-sequencing of the bacterial 16S rRNA gene V3-V4 region and fungal 18S genes V5-V7 region was conducted at Shanghai Majorbio Bio-pharm Technology Co., Ltd. to evaluate the soil microbial community structure. The primer sets 338F/806R [21] and SSU0817F/1196R [22] were used for bacterial and fungal PCR reactions. The PCR products were electrophoresed on 2% agarose gel for detection, purified and quantified with an Agarose Gel DNA Purification kit, and sequenced on an Illumina MiSeq platform (Illumina Inc., San Diego, CA, USA). Raw sequence reads were de-multiplexed, quality-filtered, and processed using QIIME (Version 1.7.0), and then clustered into operational taxonomic units (OTUs) at a 97% level of sequence similarity. The Ribosomal Database Project (RDP) Classifier were carried out for taxonomic analysis. The bacterial Silva reference (http://www.arb-silva.de, accessed on 17 August 2021) for bacterial 16S rRNA genes and the Unite reference database (http://unite.ut.ee/index.php, accessed on 17 August 2021) for fungal 18S genes were employed to annotate taxonomic information. Community composition was characterized at the phylum level. Alpha diversity (Chao and Shannon indices) of soil microbes at the OTU level was calculated with QIIME.

### 2.3. Statistical Analysis

ANOVA with Duncan’s multiple range and least significant difference (LSD) multiple range tests were employed to detect the differences in plant community, soil physicochemical properties, and microbial diversity among the three treatments. After normalization, soil bacterial and fungal community richness and diversity were investigated using the Chao and Shannon indices, respectively. The Euclidean distance was calculated to perform principle co-ordinate analysis (PCoA), which showed the dissimilarities between the microbial communities (bacteria and fungi) at the OTU level in the three soils subjected to different ground covers. Statistical differences between soil microbial communities were analyzed using PERMANOVA (permutational multivariate analysis of variance). Spearman’s correlation analyses were utilized to evaluate the relationships between soil bacterial and fungal phyla, microbial diversity, grass community properties, and soil physiochemical variables. The independent contributions of each grass and soil variable to microbial community structures were estimated through hierarchical partitioning [23]. The variables of soil bacterial and fungal community structures were quantified and derived from the axis 1 (PC1) of the above-characterized PCoA. Statistical analyses were implemented in R 4.0.5 (http://www.R-project.org, accessed on 17 August 2021). The R package “vegan” was applied for PCoA, and “hier.part” was used for hierarchical partitioning [23].

## 3. Results

### 3.1. Grasses and Soil Properties

A total of 13 grass species were detected in NG; the dominant species was *Oxalis corniculata*. Both LP and NL contained six species, with *Vicia villosa* var. *glabrescens* as dominant species (Table 1). The sowing of the legume significantly increased grass coverage as well as shoot and root biomass. Compared with NG, BC in LP and NL increased by 30–41% and 60–67%, and BN by 70–76 and 82–91%, respectively. The δ^15^N values of the N_2_-fixing legume species significantly differed from those of the reference plants, allowing the calculation of the proportion of the N derived from symbiotic fixation. *Vicia villosa* var. *glabrescens* fixed high proportions of N (44–52%), and the annual amounts of N fixed in LP and NL were 5.71 and 5.01 g m^−2^, respectively.

Although no significant difference was found in soil bulk density among the treatments, 6 years after the establishment of the experiment, the sowing of the legume resulted in an obvious improvement in soil fertility when compared with natural grass monoculture (Table 2). Besides pH, all determined soil chemical properties were significantly increased in LP and NL compared to NG. However, the difference between LP and NL was considerably lower than that between treatments with and without legume sowing.

### 3.2. Soil Microbial Community Composition and Diversity

A total of 486,078 and 431,883 valid sequences were obtained for bacteria and fungi, respectively. After OTU removal of 97% similarity, 4841 bacterial OTUs from 33 phyla were detected, and 320 fungi OTUs from 31 phyla were classified (Figure S1).

**Figure 1:**
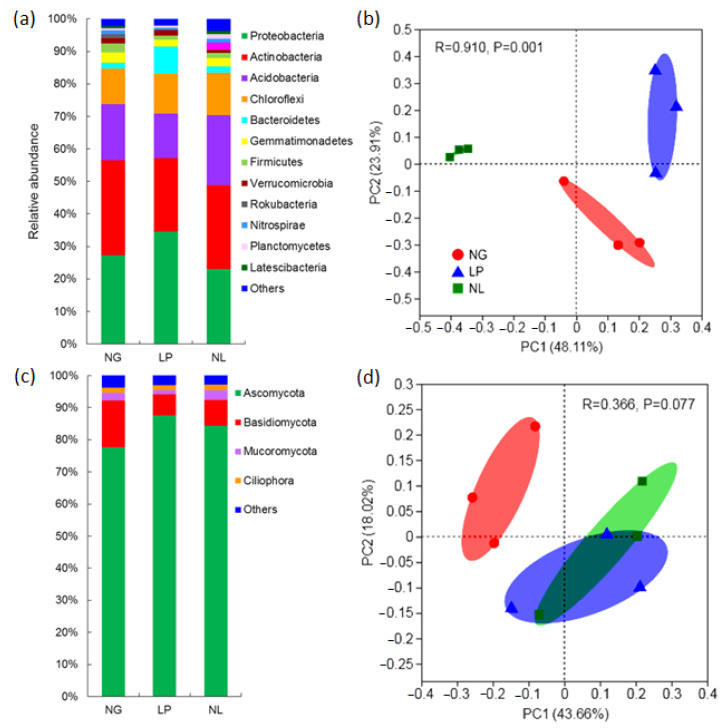
Relative average abundances of the dominant phyla (>1%) of (**a**) bacteria and (**c**) fungi in soils, and PCoA of changes in soil bacterial (**b**) and fungal (**d**) communities based on OTU richness. In (**b**,**d**), the R value is the degree of interpretation of the sample difference. The actual range of the R value is (−1, 1), generally between (0, 1), R > 0, indicating a difference between the groups; generally, R > 0.75: Large difference; R > 0.5: medium difference, R > 0.25: small difference. R equal to 0 or near 0 indicates that there is no difference between the groups. The *p*-value indicates the significance of grouping. Generally, *p* < 0.05 means statistical difference and *p* < 0.01 means significant difference.

The dominant bacterial phyla were *Proteobacteria*, *Actinobacteria*, and *Acidobacteria*, accounting for 68.0% of the total bacterial abundance. Their individual relative abundances were 23.55–34.61%, 22.58–29.46%, and 13.71–21.97%, respectively, followed by *Chloroflexi* (11–13.38%), *Bacteridetes* (1.69–8.32%), *Gemmatimonadetes* (2.22–3.36%), *Firmicutes* (1.23–2.66%), and *Verrucomicrobia* (1.12–1.70%) (Figure 1a). Compared to NG and NL, LP showed a significantly higher relative abundance of *Proteobacteria* and *Bacteroidetes* but a significantly lower relative abundance of *Actinobacteria* and *Acidobacteria*. Non-significant difference was found for the relative abundance of dominant phyla between NG and NL. A deeper taxonomic analysis demonstrated a significant effect of legume sowing on the phylum *Proteobacteria*, *Actinobacteria*, and *Acidobacteria* (Figure S2a). A significant clustering pattern was found where between groups variation was much higher compared to within group variation for bacteria (Figure 1b), indicating the dissimilarity of bacterial communities among treatments.

Among the fungal phyla, *Ascomycota* was dominant, with a relative abundance of 78.91–88.29%, followed by *Basidiomycota* and *Mucoromycota* with 6.54–14.66% and 1.27–3.11%, respectively (Figure 1c). Compared with NG, ground covering with the legume led to a significant increase in the relative abundance of *Ascomycota* and a significant decrease in the relative abundance of *Basidiomycota*. A deeper taxonomic analysis demonstrated that two genera’s relative abundances (one in Sordariomycetes, one in Dothideomycetes) in phylum *Ascomycota* were significantly increased, and two genera’s relative abundances (two in Tremellomycetes) in phylum *Basidiomycota* were significantly decreased by the legume sowing (Figure S2b). Non-significant difference was found for the relative abundance of the dominant phyla between LP and NL. For Figure 1d, pairwise comparisons between individual groups would likely be informative as it appears that LP and NL would be significantly different from NG but not from each other. In general, β-diversity analysis (based on OTU frequencies) of the bacterial and fungal communities (Figure 1b,d) shows greater differences in composition of the bacterial communities compared to the fungal communities, which may due to the abundance differentials at the OUT level between each treatment (Figure S3).

**Figure 2:**
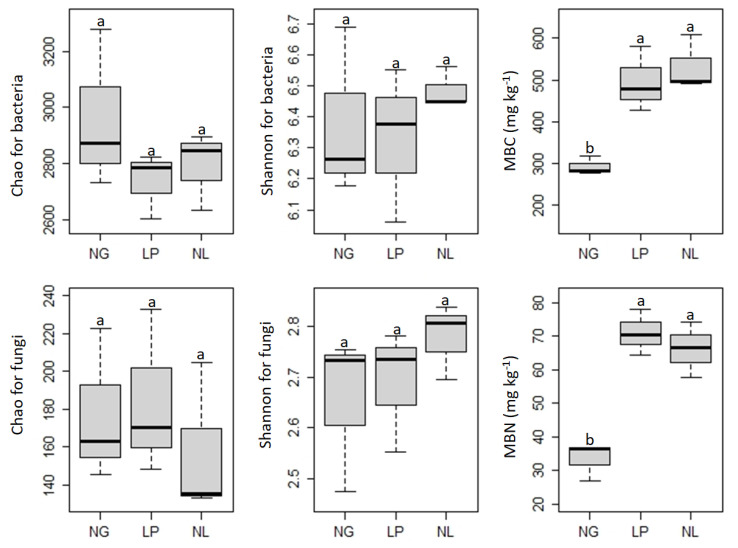
Effects of ground coverage on Chao and Shannon indices for soil bacterial and fungal communities, MBC, and MBN in citrus orchards. MBC: microbial biomass carbon, MBN: microbial biomass nitrogen. Different lowercase letters indicate significant differences (*p* < 0.05).

In general, ground covering with *Vicia villosa* var. *glabrescens* slightly decreased community richness but increased community diversity when compared with NG. However, there was no significant difference between community richness and diversity for bacteria and fungi among the treatments (Figure 2). Compared with NG, ground covering with *Vicia villosa* var. *glabrescens* significantly increased soil microbial biomass C and N, but no significant difference was detected between LP and NL (Figure 2).

### 3.3. Responses of Soil Microbial Community and Biomass to Grass and Soil Properties

**Figure 3:**
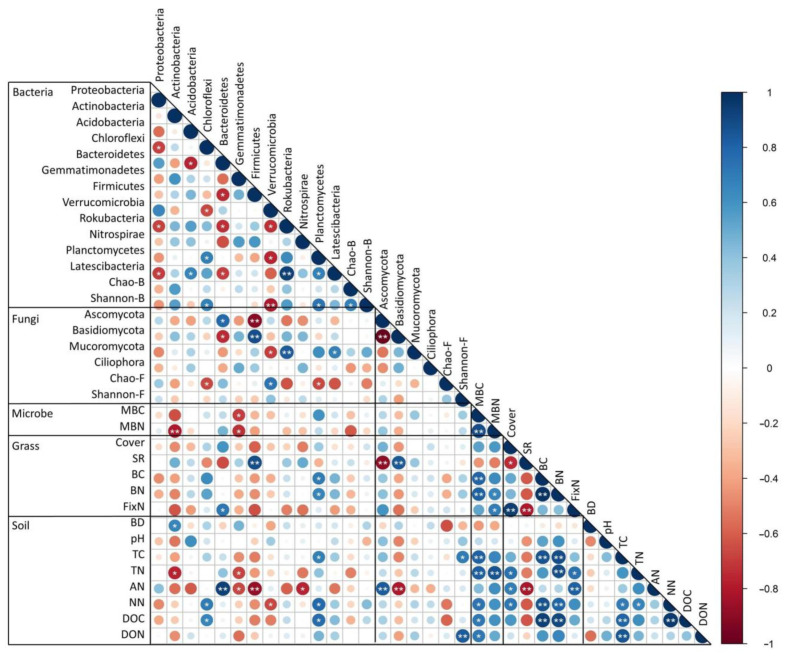
Relationship between grass communities, soil properties, and microbial communities. Cover: Plant coverage; Height: Grass average height; SR: Grass species richness; BC: Biomass C; BN: Biomass N; FixN: Plant symbiotic N fixation; BD: soil bulk density; TC: soil total carbon; TN: soil total nitrogen; AN: NH_4_^+^-N, NN: NO_3_^−^-N; DOC: dissolved organic carbon; DON: dissolved organic nitrogen; MBC: microbial biomass carbon; MBN: microbial biomass nitrogen. *: *p* < 0.05, **: *p* < 0.01.

Grass biomass C and N, which were mainly determined by grass coverage and height, showed a significant (*p* < 0.01) positive relationship with soil TC, NN, and DOC levels (Figure 3). In addition, plant symbiotic N fixation showed a significant positive relationship with soil TN (*p* < 0.05) and AN (*p* < 0.01).

Grass biomass and plant symbiotic N fixation greatly affected soil microbial biomass. Significant positive relationships between BC, BN, and MBC (*p* < 0.01) as well as between BN, FixN, and MBN (*p* < 0.05) were observed. However, the impact of grasses on dominant soil microbial phyla was limited, and a significant relationship was only detected between FixN and the relative abundance of Bacteroidetes (*p* < 0.05) as well as between BC, BN, and *Planctomycetes* abundance (*p* < 0.05).

In general, both soil TC and TN showed a consistent and significant positive correlation with soil microbial biomass, whereas the relationship between soil chemical properties and dominant soil microbial phyla was inconsistent. Soil AN was significantly positively related with the relative abundances of *Bacteroidetes* (*p* < 0.01) and *Ascomycota* (*p* < 0.01), but significantly negatively related with the relative abundances of *Gemmatimonadetes* (*p* < 0.05), *Firmicutes* (*p* < 0.01), *Nitrospirae* (*p* < 0.05), and *Basidiomycota* (*p* < 0.01). Soil TN showed significant negative relationships with the relative abundances of *Actinobacteria* (*p* < 0.01) and *Gemmatimonadetes* (*p* < 0.01), whereas NN and DOC were significantly positively related to the relative abundances of *Chloroflexi* (*p* < 0.01) and *Planctomycetes* (*p* < 0.01).

**Figure 4:**
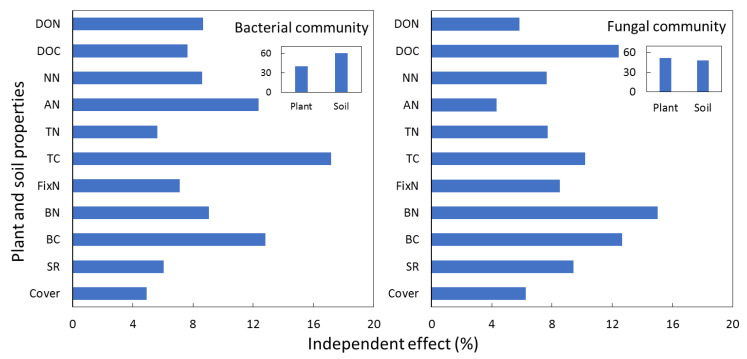
Hierarchical partitioning of grass and soil factors explaining variations in the soil microbial community.

Six soil factors and five plant factors were incorporated into hierarchical partitioning analysis. Soil factors accounted for 60.1% of the independent effects on the soil bacterial community, of which TC was the most influential factor and accounted for 17.0% of the total impact (Figure 4). Further, BC and AN accounted for 12.8 and 12.1% of the total factors. Soil and plant factors contributed equally to the variations in the fungal community and microbial biomass. The factor BN accounted for 15.0% of the factors explaining soil fungal community, followed by DOC, accounting for 12.4%.

## 4. Discussion

### 4.1. Grass Biomass Is a Strong Driver of Soil Chemical Properties

Planting grass has been proven to improve soil quality [24], while the present study further indicated that sowing of legumes resulted in an obvious improvement on soil nitrogen content than the natural grass monoculture. In addition, grass biomass showed a significant (*p* < 0.01) positive relationship with most determined soil chemical properties, indicating that grass biomass is a strong driver of soil chemical properties. In the present study, *Oxalis corniculata* was the dominate species in NG, whereas *Vicia villosa* var. *glabrescens* was the dominate species in NL and LP. Compared with *Oxalis corniculata*, which is a “hitchhiker” in plots, *Vicia villosa* var. *glabrescens*, which was selected as a ground cover species, showed a better adaptability to the microenvironment of the orchard, resulting in greater biomass accumulation and organic matter return. Although the differences in annual grass biomass C among the plots were negligible when compared to those in the soil native C pool, the accumulated C after 6 years was estimated at 648 g m^−2^ and the accumulation of soil nutrients in NL and LP can be explained by organic matter input via returning grass biomass to the soil [25].

Legumes can increase N availability in soils by fixing atmospheric N [26]. Symbiotic N fixation by *Vicia villosa* var. *glabrescens* in the present study accounted for more than 1/3 of the biomass N in NL and LP. The fixed N can be transferred to co-occurring plants via root exudation [27] and decomposition of dead tissue [28]. In our study, about 5.01–5.71 g m^−2^ of extra N was added annually in NL and NP plots by the biomass of legumes when compared with NG, in which all of the biomass N was derived from soil. As the addition of chemical fertilizer did not differ among the treatments, symbiotic N fixation was the main factor increasing the soil nitrogen levels throughout the experimental period, and a significant positive relationship between FixN and soil AN (*p* < 0.01), TN (*p* < 0.05) was detected.

Generally, decreased grass-legume ratios may initially enhance BNF due to increased legume density [29]. However, no significant difference in symbiotic N fixation between NL and LP was observed in this study, which implied that tripled seeding rates of legumes only had a limited effect on biological N fixation (BNF) rates. Li et al. [26] indicated that a legume-grass ratio of 4:4 results in a higher BNF rate than 0:4, 4:0, and 3:1. Although the competition of grasses for nitrogen in the NL can improve BNF efficiency [30], fierce intraspecific competition between legume species in LP may result in less interaction between grasses and legumes in terms of N use, which may inhibit BNF via legumes [26].

Among the various factors driving soil structural changes, plant roots were reported to play important roles in creating, exploiting, and occupying the soil pore space [31]. However, significant differences in root biomass among the treatments and non-significant differences in bulk density were found in the studied plots. Indeed, the bulk density of 1.42–1.44 g cm^−3^ in the present study was higher than that reported for most agricultural systems. This phenomenon may be attributed to the no-tillage practice in orchards, and the effect of grass roots on soil bulk density was eliminated by compaction beneath agricultural machinery. Overall, our results partly support the first hypothesis that grass biomass impacts soil chemical properties, but not physical properties.

### 4.2. Ground Cover and Soil Properties Both Significantly Impact the Soil Microbial Community

Compared with NG, the sowing of *Vicia villosa* var. *glabrescens* in LP and NL significantly increased soil microbial biomass. Similar results have been reported by Breulmann et al. [32] and Stephan et al. [33], where monocultures of legumes and mixed cultures of non-leguminous herbs and legumes showed improved soil microbial biomass and activities compared to grasses. Microbial biomass growth could be stimulated by the increased availability of nutrients from returned ground cover [34]. The significantly positive correlations of soil MBC and MBN with grass BC, BN, and FixN support this assumption.

The distance between microbial community centroids of NG from NL and LP in the PCoA indicates differences in the soil microbial community structure between plots with and without legumes. Similar results have been reported by Fox et al. [35], who further suggested that the physiological differences among grass species, especially the release of symbiotically-fixed N_2_ from legume species, may be an important driver of the microbial community structure. However, the hierarchical portioning results in this study revealed that soil properties contributed equally or even more than grass properties to the soil microbial community, and FixN only showed a significant relationship with the relative abundance of Bacteroidetes (*p* < 0.05), explaining less than 9% of the soil bacterial and fungal communities. This does not support our second hypothesis, mainly because (1) all plots received chemical N fertilization equal to 410 kg N ha^−1^ yr^−1^, which was considerably higher than the amount of biologically fixed N (about 50–57 kg N ha^−1^ yr^−1^), and thus, the effect of FixN on the soil microbial community was eliminated; (2) the soil properties override the plant effects, and soil microbial communities are strongly shaped by soil nutrient contents [36].

The significant relationships of most dominant microbial phyla (10 out of 16) with the soil nitrogen content (TN, AN, or NN) indicate that soil nutrients prominently influenced soil microbial richness and abundance. Some abundant bacterial phyla can be divided into broad ecological categories that correspond to copiotrophic and oligotrophic groups. *Acidobacteria* is the most abundant bacterial phylum in soils with low resource availability, whereas β-*Proteobacteria* and *Bacteroidetes* exhibited copiotrophic attributes favored by nutrient-rich conditions [37,38,39]. In the present study, the highest relative abundances of *Proteobacteria* and *Bacteroidetes* were found in LP soil with the highest TN and AN contents. Undoubtedly, significant positive relationships (*p* < 0.05) between soil AN, NN, and relative abundances of *Proteobacteria* and *Bacteroidetes* were observed. We also discovered a higher relative abundance of *Ascomycota* and a lower relative abundance of *Basidiomycota* in NL and LP soils than those in NG, as well as a significant positive relationship (*p* < 0.01) between soil TN and the relative abundance of *Ascomycota*. Weber et al. [40] also found an increased relative abundance of *Ascomycota* and a decreased relative abundance of *Basidiomycota* in N-rich soils. Although soil N showed significant correlations with the abundance of multitudinous dominant microbial phyla, none of the determined N forms (TN, AN, NN, and DON) were the most influential factor driving the microbial community. The reasons might be as follows: (1) the responses of microorganisms to different N sources were varied and complex, and (2) the response of dominant microbial phylum abundances cannot represent the whole microbial community structure.

It is essential to better understand the causes and controls of soil microbial composition since soil microbial communities play an essential role in regulating carbon and nitrogen cycling as well as mineralization and stabilization [10]. Various studies at the plot scale have found diverse biotic and abiotic factors including soil type and properties, aboveground biotic diversity, density, and physiological differences among species and microbial successional stages, which significantly influenced soil microbial composition and structure [35,36,41]. Nonetheless, the main controlling factors are still largely unclear. Fox et al. [35] and Hammelehle et al. [36] indicated that the physiological differences among grass species drive microbial community structures. In contrast, Marschner et al. [42] indicated that soil microbial communities are strongly shaped by soil properties, whereas the plant species composition of the grasslands was less important [43]. Hierarchical partitioning analysis in the present study showed that soil properties and ground cover properties almost equally contributed to the soil microbial community. It is noteworthy that microbial community construct was an integrated effect of all influencing factors, and the major determinant of the microbial community may depend on the magnitude of variation in these factors. In this sense, ground cover properties may contribute more significantly to the soil microbial community during the initial stage of a long-term experiment, as the soil properties of each plot were at the same state. After several years, the varied soil properties would eliminate the effect of ground cover, making them the most influential factors driving the microbial community. Further long-term studies are needed to confirm this assumption and to clarify the dynamics of soil microbial structure over time.

## Figures and Tables

**Table 1:** Characteristics of grass communities in different treatments.

Properties	NG	LP	NL
Dominant species (coverage)	*Oxalis corniculata* (25%) *Duchesnea indica* (25%) *Veronica didyma* (11%) *Echinochloa crusgalli* (6%)	*Vicia villosa* var. *glabrescens* (80%) *Duchesnea indica* (12%) *Galium aparine* (8%)	*Vicia villosa* var. *glabrescens* (30%) *Echinochloa crusgalli* (8%) *Duchesnea indica* (7%) *Galium aparine* (7%)
Grass coverage (%)	71 b	94 a	89 a
SR	13 a	6 b	6 b
SB (kg m^−2^)	0.24 b	0.32 a	0.36 a
RB (kg m^−2^)	0.17 c	0.24 b	0.33 a
BC (g m^−2^)	170.08 c	230.71 b	278.19 a
BN (g m^−2^)	8.72 b	15.10 a	16.42 a
FixN (g m^−2^)	0 c	5.71 a	5.01 a

Different lowercase letters indicate significant differences (*p* < 0.05); dominant species with coverage higher than 5% are shown. SR: Grass species richness; SB: Shoot biomass; RB: Root biomass; BC: Amount of grass biomass accumulated C; BN: Amount of grass biomass accumulated N; FixN: Amounts of plant symbiotic N fixation.

**Table 2:** Soil physicochemical properties under different ground cover layers in citrus orchards.

Soil Property	NG	LP	NL
BD (g cm^−3^)	1.44 a	1.42 a	1.42 a
pH	6.03 a	6.08 a	6.13 a
TC (g kg^−1^)	5.09 c	9.84 b	13.33 a
TN (g kg^−1^)	0.83 b	1.26 a	1.22 a
AN (mg kg^−1^)	1.97 c	2.91 a	2.28 b
NN (mg kg^−1^)	15.02 b	19.32 a	20.16 a
DOC (mg kg^−1^)	13.73 b	21.12 a	25.63 a
DON (mg kg^−^^1^)	26.09 b	36.04 ab	47.88 a

Different lowercase letters indicate significant differences (*p* < 0.05). BD: soil bulk density; TC: soil total carbon; TN: soil total nitrogen; AN: NH_4_^+^-N, NN: NO_3_^−^-N; DOC: dissolved organic carbon; DON: dissolved organic nitrogen.

## Data Availability

Not applicable.

## Supplementary Materials

The following are available online at https://www.mdpi.com/article/10.3390/microorganisms9122507/s1, Figure S1: OTU Venn diagram of soil bacteria (a) and fungi (b) with different treatments; Figure S2: Relative abundances of genera in dominate phyla of bacteria (a) and fungi (b) in soils under different ground covers; Figure S3: Abundance differentials at the OUT level of bacteria (a) and fungi (b) among different ground cover treatments based on Kruskal-Wallis H test
