# Supplementary material for: Responses of Soil Microbial Traits to Ground Cover in Citrus Orchards in Central China
Source: Microorganisms. 2021 Dec 3;9(12):2507. doi: 10.3390/microorganisms9122507 (PMC8708208; doi:10.3390/microorganisms9122507)
Supplement: Supplementary file 1 [file microorganisms-09-02507-s001.zip › microorganisms-1452920-supplementary.pdf]

Supplementary Materials

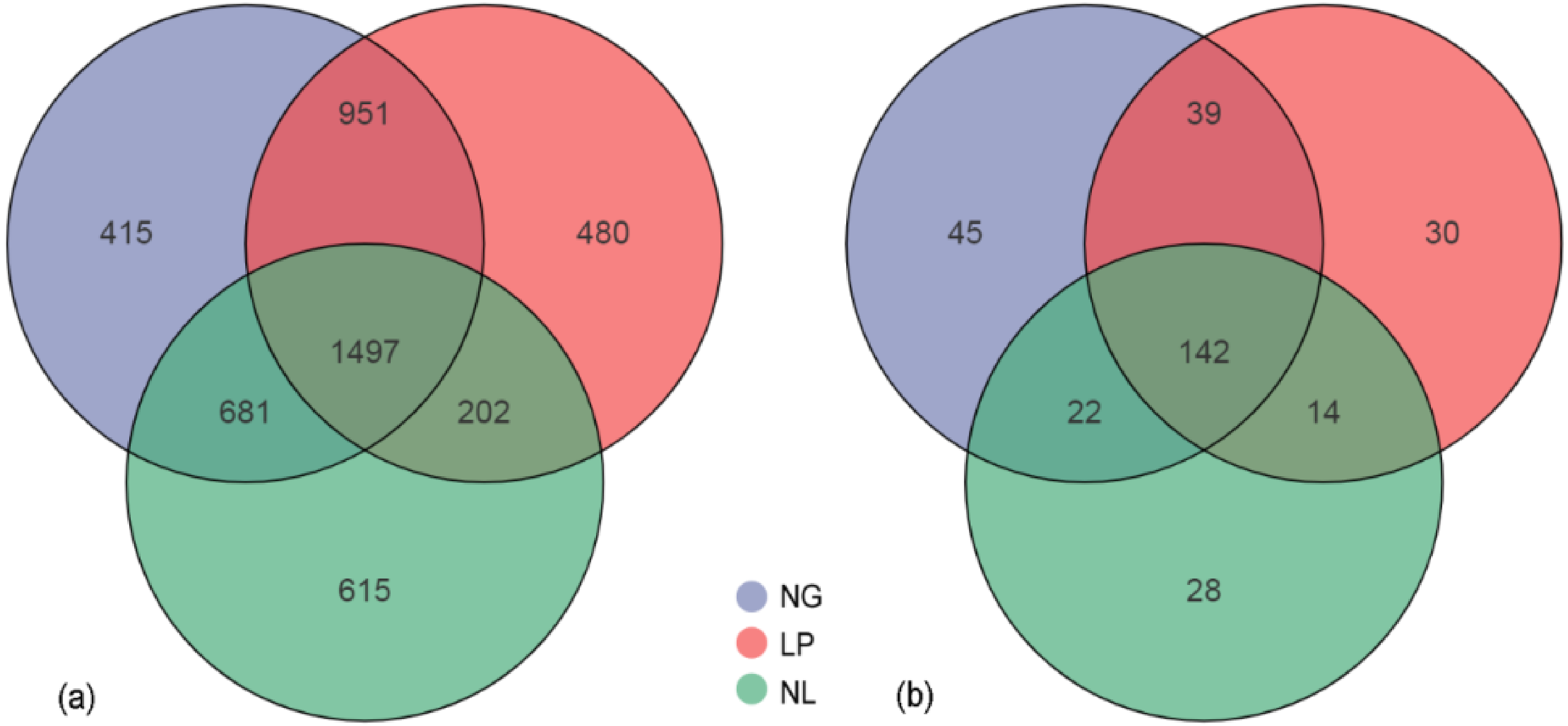

Figure S1. OTU Venn diagram of soil bacteria (a) and fungi (b) with different treatments.

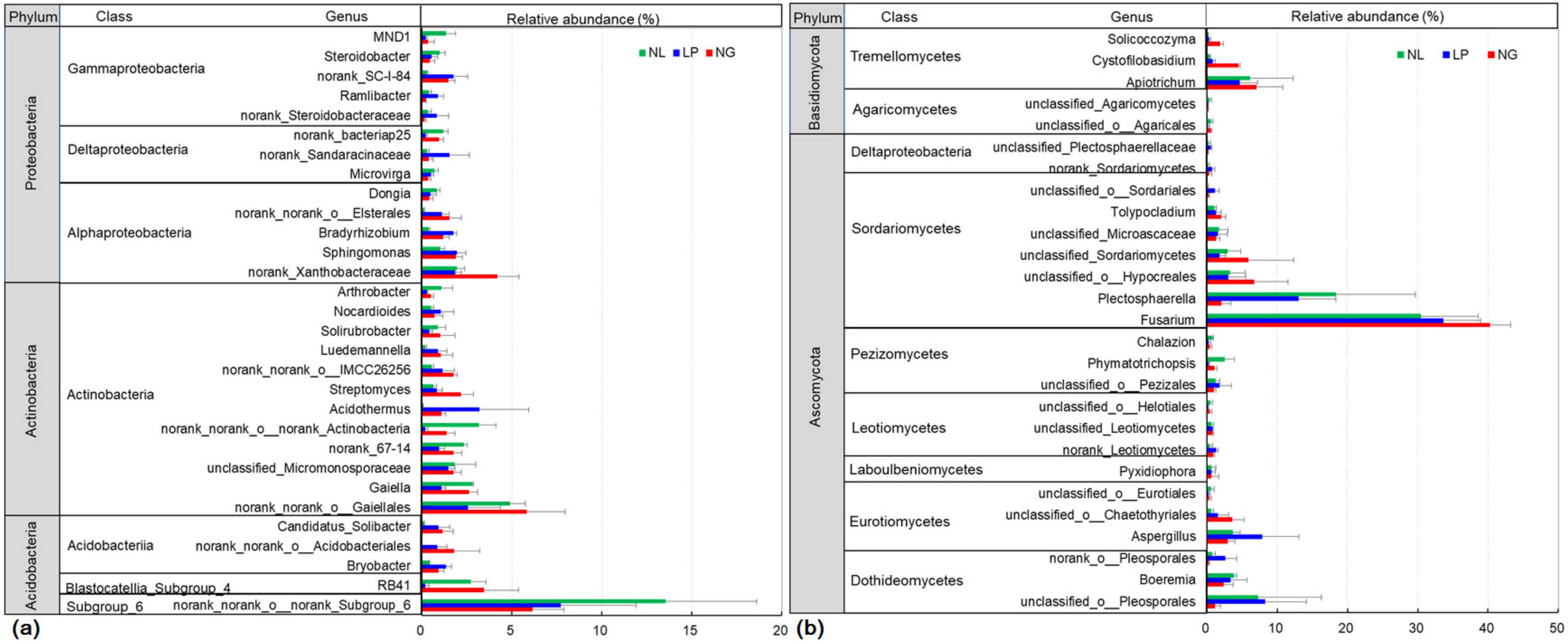

Figure S2. Relative abundances of genera in dominate phyla of bacteria (a) and fungi (b) in soils under different ground covers.

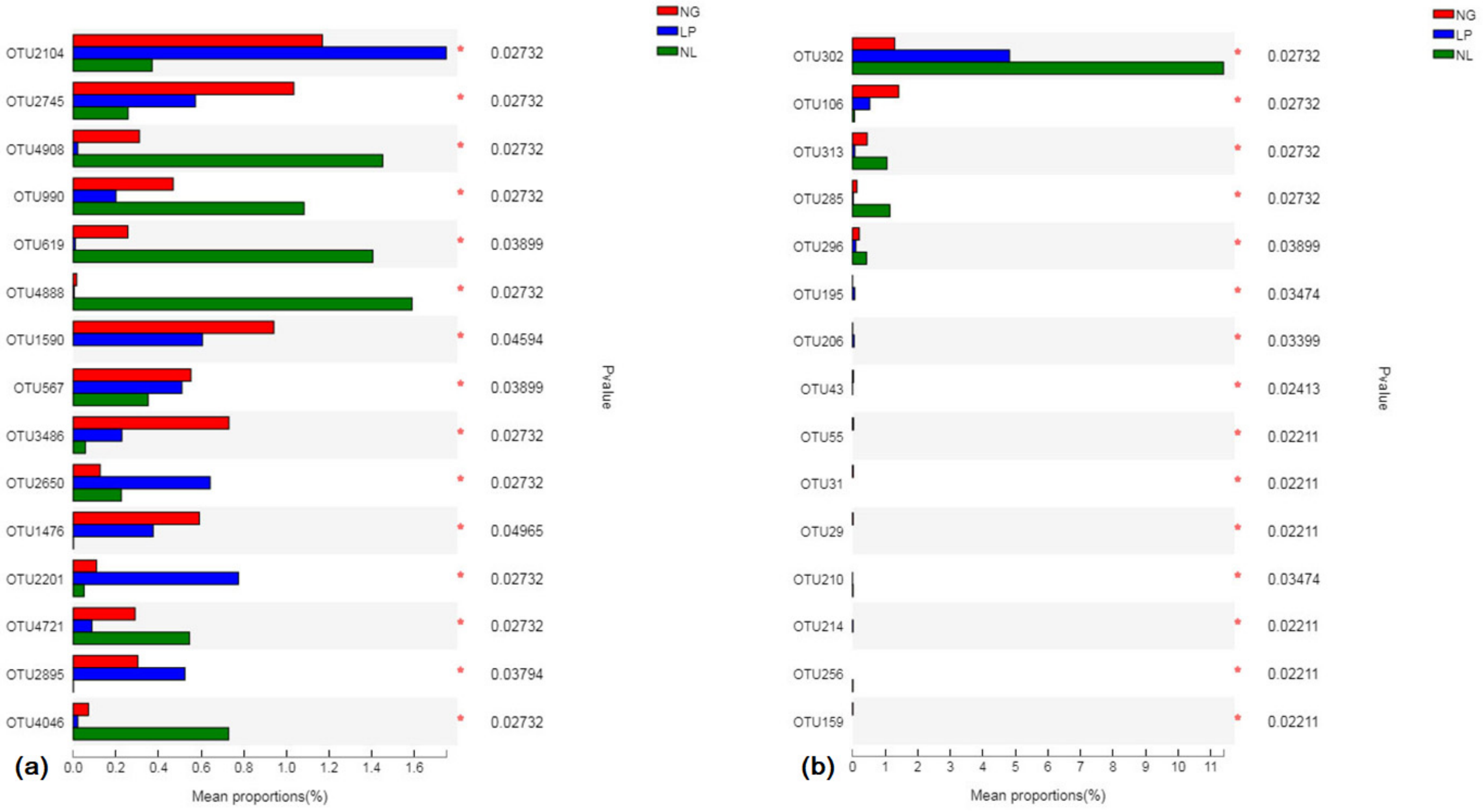

Figure S3. Abundance differentials at the OUT level of bacteria (a) and fungi (b) among different ground cover treatments based on Kruskal-Wallis H test
